# A Prospective Study on Assessment of Microbial Contamination of Toothbrushes and Methods of Their Decontamination

**DOI:** 10.7759/cureus.30155

**Published:** 2022-10-10

**Authors:** Sasi Pradeep, Gopu Nandini, Sunkara Hiranmayi, Goutham Kumar, Nikhil K Bijjala, Spandana Guduri

**Affiliations:** 1 Department of Oral Pathology and Microbiology, Sai Krishna Dental Clinic and Hospital, Khaleelwadi, IND; 2 Department of Public Health Dentistry, Meghna Institute of Dental Sciences, Nizamabad, IND; 3 Department of Oral and Maxillofacial Surgery, Government Dental College and Hospital, Hyderabad, IND; 4 Department of Oral Pathology and Microbiology, Government Dental College and Hospital, Hyderabad, IND; 5 Department of Prosthodontics, Government Dental College and Hospital, Hyderabad, IND

**Keywords:** dettol, listerine, chlorhexidine, decontamination, toothbrushes, microbial contamination

## Abstract

Background: Toothbrushes may get contaminated by the oral cavity, environment, hands, storage containers, or aerosol contamination. The present study was conducted to assess the microbial contamination of toothbrushes and methods of their decontamination.

Materials and methods: The current study included 160 subjects of both genders. All the subjects were provided with a toothbrush and paste with complete hygiene instructions for the oral cavity. After one month, all the brushes were collected. The samples were categorized into four groups of 40 each. Group I was treated with 0.2% chlorhexidine gluconate, group II with Listerine, group III with Dettol, and group IV with tap water. Finally, these toothbrushes were placed in 5 mL of neutralizer broth and then evaluated to study the efficacy of four disinfectants. All the data were analyzed using the statistical package for social science (SPSS) version 23 software (IBM, Armonk, NY, USA). For all analyses, p < 0.05 was considered to be statistically significant

Results: Aerobic bacterial growth before disinfection in Groups I, II, III, and IV was 91.6%, 75.84%, 75%, 81.67%, respectively (p = 0.01). After disinfecting the brushes aerobic bacterial growth was reduced to 34.17%, 30.84%, 24.17% & 74.17% in Groups I, II, III, and IV, respectively (p = 0.002). Klebsiella, Micrococci and Escherichia coli survived the most even after disinfection was done.

Conclusion: Most effective agent for the disinfection of toothbrushes was Dettol followed by Listerine and 0.2% chlorhexidine gluconate. Tap water was found to be ineffective in the decontamination of toothbrushes.

## Introduction

Oral health is the gateway to systemic health and the overall well‑being of people. The oral microbiome consists of various bacteria, viruses, and fungi that are responsible for causing a number of oral diseases [1]. Proper oral hygiene maintenance can greatly reduce these microbes and help in achieving oral health. The toothbrush is the most widely used mechanical means of maintaining good oral hygiene. However, toothbrushes often get contaminated with use and improper storage [2].

Toothbrushes are generally stored in a common container in the bathroom, which can introduce microbes to the toothbrush. Toothbrush infected from the unhygienic oral cavity, surroundings, hands, or storage site [3]. Bacteria that attach, accumulate & multiply on toothbrushes may be transferred to the oral cavity leading to different diseases. Thus, it is essential to frame uniform oral health guidelines to prevent toothbrush contamination [4,5]. Contaminated toothbrushes may broadcast microorganisms, which could be detrimental to oral and systemic health [6].

In the general public, a lack of awareness was observed regarding the maintenance and care of toothbrushes. An extensive literature search revealed many studies regarding the cleaning of toothbrushes, but no clear method of disinfection was observed. Therefore, a uniform and ideal cost-effective method is required for proper care of toothbrushes that can culminate in the risk of microbial transmission in the oral cavity. The present study was conducted to assess the microbial contamination of toothbrushes and methods of their decontamination.

## Materials and methods

The current study included 160 subjects of both genders. The subjects were enrolled only after taking written consent. The study was carried out in Sai Krishna Dental Clinic and Hospital, Khaleelwadi, Nizamabad, Telangana, during the period January 2022 to June 2022. Ethical clearance was obtained (ethical number- SKDC/NZM/Exp/2021-22). The demographic profile of subjects was recorded such as name, age, gender, etc.

The study includes subjects of age 18-45 years with a gingival index score of two or three with at least 20 natural, unbroken teeth. The subjects on any type of treatment like antibiotics, anti-fungal, antimicrobials, any history of dental surgery or patients visiting the clinic and not taking treatment, smoking, and systemic diseases were excluded from the study.

The samples were categorized into four groups of 40 each. Group I was treated with 0.2% chlorhexidine gluconate, group II with Listerine, group III with Dettol, and group IV with tap water. All the subjects were provided with toothbrushes and paste with complete hygiene instructions for the oral cavities. Five unused new toothbrushes (negative control) were also kept along with them. After a period of one month, all the brushes were collected. They were kept in sterile boxes. The samples were transported for microbial analysis.

Firstly, the bristle part of toothbrushes was immersed in test tubes containing 5 milliliters of normal saline for one hour. After that, they were cultured in Mueller-Hinton‑based blood agar and MacConkey agar. They were incubated at 37°C for 24 h, aerobically. Identification of isolates was done by Gram staining. Further, 1 mL of the sample was also injected into Robertson’s cooked meat (RCM) medium (temperature 37°C for 24 h). Following this, it was further cultured in Mitis Salivarius agar.

Each toothbrush was then disinfected by immersing it in one of the four disinfectants for one hour. After one hour, these toothbrushes were placed in 5 mL of neutralizer broth, and then the samples from the neutralizer broth were collected and cultured to evaluate the efficacy of the four disinfectants used in the study. The evaluation was done by observing the growth of microorganisms after the treatment of disinfectant.

Statistical analysis

A total of 160 participants were selected, which is derived after taking 5% Type I error and standard normal variate for power as 90%. The mean proportion of reduction is considered 70% which includes 40 patients in each group. All the data were analyzed using the statistical package for social science (SPSS) version 23 software (IBM, Armonk, NY, USA). Inferential statistics were performed using Chi‑square analysis. For all analyses, p < 0.05 was considered to be statistically significant.

## Results

Table 1 depicts the aerobic bacterial growth distribution in the four test groups before disinfection after using it for a month. Out of 120 agar plates in each group, there was statistically significant aerobic bacterial growth noted in groups I, II, III, and IV at 91.6%, 75.84%, 75%, and 81.67%, respectively (p = 0.01). All the five new brushes included as control showed negative growth.

**Table 1:** Assessment of aerobic bacterial growth before disinfection *Statistically significant. Data presented as Number (Percentage)

Groups	Percentage of aerobic bacteria		P-value
	Present	Absent	
Group I	110 (91.6)	10 (8.4)	0.01*
Group II	91 (75.84)	29 (24.16)	
Group III	90 (75)	30 (25)	
Group IV	98 (81.67)	22 (18.33)	

After immersing the toothbrushes in disinfectants for one hour, there was a significant reduction (p = 0.002) in bacterial growth. After disinfection, the aerobic bacterial growth was observed as 34.17%, 30.84%, and 24.17% in groups I, II, and III, respectively. However, a significant change in bacterial growth was observed after disinfection in the tap water group (p=0.002) (Table 2).

**Table 2:** Distribution of aerobic bacterial growth among the four test groups after disinfection *Statistically significant. Data presented as Number (Percentage)

Groups	Percentage of aerobic bacteria		P-value
	Present	Absent	
Group I	41 (34.17)	79 (65.83)	0.002*
Group II	37 (30.84)	83 (69.16)	
Group III	29 (24.17)	91 (75.83)	
Group IV	89 (74.17)	31 (25.83)	

**Figure 1:**
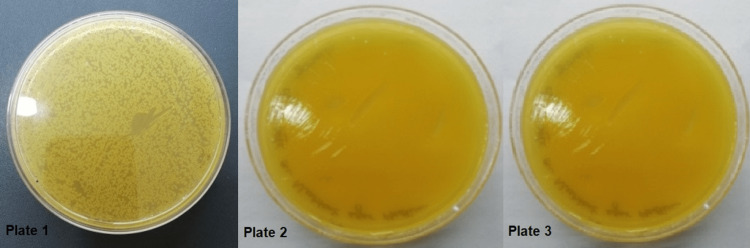
Plate 1 (Growth of Streptococcus); plate 2 (Growth of Klebsiella); plate 3 (Growth of Klebsiella)

Table 3 depicts that Micrococci and Escherichia coli comprised 25.83% and 20% of the organisms isolated from Group I. In groups II and III, the maximum colonies were observed of E. coli. However, 24.16% and 25% of culture plates had no growth in groups I and II, respectively. In group IV, 22.5% of the organism isolated from the culture media plates was Klebsiella while 18.33% of culture media showed no growth (Table 3). Figure 1 depicts culture plates showing the growth of micro-organisms.

**Table 3:** Distribution of different microorganisms before disinfection Data presented as Number (Percentage)

Microorganisms	Group I	Group II	Group III	Group IV	Total
Beta‑hemolytic streptococci	8 (6.66)	7 (5.83)	7 (5.83)	8 (6.66)	30 (6.25)
Bacillus species	11 (9.16)	8 (6.66)	10 (8.33)	6 (5)	35 (7.29)
Pseudomonas	2 (1.66)	1 (0.83)	0 (0)	0 (0)	3 (0.62)
Escherichia coli	24 (20)	21 (17.5)	18 (15)	15 (12.5)	78 (16.25)
Micrococci	31 (25.83)	18 (15)	15 (12.5)	20 (16.66)	84 (17.5)
Klebsiella	13 (10.83)	11 (9.16)	21 (17.5)	27 (22.5)	72 (15)
Streptococcus mitis	14 (11.66)	16 (13.33)	11 (9.16)	9 (7.5)	50 (10.41)
Viridans Streptococci	7 (5.83)	10 (8.33)	8 (6.66)	13 (10.83)	38 (7.91)
No growth	10 (8.33)	29 (24.16)	30 (25)	22 (18.33)	91 (18.95)
Total	120 (100)	120 (100)	120 (100)	120 (100)	480 (100)

Table 4 depicts the percentage of microorganisms found in different groups after applying disinfectants for one hour. The micro-organisms observed in the culture after disinfection included E. coli, Bacillus, Klebsiella, Beta‑hemolytic streptococci, Viridans streptococci, Streptococcus mitis, and micrococci (Table 4). Out of all microorganisms, 9.58% of them were Klebsiella and more than 8% were E. coli and micrococci (Table 4).

**Table 4:** Distribution of different microorganisms after disinfection Data presented as Number (Percentage)

Microorganisms	Group I	Group II	Group III	Group IV	Total
Beta‑hemolytic streptococci	2 (1.66)	0 (0)	0 (0)	5 (4.16)	7 (1.45)
Bacillus species	4 (3.33)	3 (2.5)	2 (1.66)	5 (4.16)	14 (2.91)
Pseudomonas	0 (0)	0 (0)	0 (0)	0 (0)	0 (0)
Escherichia coli	11 (9.16)	10 (8.33)	7 (5.83)	14 (11.66)	42 (8.75)
Micrococci	10 (8.33)	5 (4.16)	6 (5)	18 (15)	39 (8.12)
Klebsiella	7 (5.83)	7 (5.83)	8 (6.66)	24 (20)	46 (9.58)
Streptococcus mitis	6 (5)	9 (7.5)	5 (4.16)	11 (9.16)	31 (6.45)
Viridans Streptococci	1 (0.83)	3 (2.5)	1 (0.83)	12 (10)	17 (3.54)
No growth	79 (65.83)	83 (69.16)	91 (75.83)	31 (25.83)	284 (59.16)
Total	120 (100)	120 (100)	120 (100)	120 (100)	480 (100)

## Discussion

Toothbrush contamination is an unavoidable outcome of use and improper storage resulting in many systemic and oral infections [7]. With regular use, the bristles often get worn out. Hence, the American Dental Association recommends replacing toothbrushes at an interval every three to four months [8]. But this recommendation does not clearly mention if the replacement of the toothbrush could also help avoid microbial contamination [9]. Secondly, more frequent changes in toothbrushes could pose an economic burden. Routine household procedures such as rinsing and drying seem to be a good method but might not be sufficient to reduce the microbes [10,11]. The present study was conducted to assess the microbial contamination of toothbrushes and methods of their decontamination.

We found that aerobic bacterial growth before disinfection was 91.6%, 75.84%, 75%, and 81.67% in groups I, II, III, and IV, respectively. Joy et al. assessed the microbial toothbrush contamination and their decontamination using different disinfectants by involving 80 subjects with two or three gingival index scores [12]. Toothbrushes were divided into four groups of 20 subjects each. Group A was treated with 0.2% chlorhexidine gluconate, group B with Listerine, group C with Dettol, and group D with tap water for 1 h. All the toothbrushes, which were sampled had significant bacterial growth after one month of use. Similar to our study Joy et al. found a maximum of the toothbrushes were contaminated with E. coli (22.7%). All the tested disinfectants significantly reduced bacterial growth and Dettol showed maximum effectiveness. We found that aerobic bacterial growth after disinfection was present in 34.17%, 30.84%, 24.17%, and 74.17% in groups I, II, III, and IV, respectively. Caudry et al. in their study also found that toothbrushes are profoundly contaminated with normal use. Moreover, they observed that disinfection with tap water had not given appreciable results [13].

Mehta et al. found that 70% of the toothbrushes in their study got greatly contaminated with pathogenic microorganisms after their use [14]. According to them, Beta‑hemolytic streptococci/Bacillus species were seen in 20% of samples, Pseudomonas/Bacillus in 15%, and coagulase‑negative staphylococci, Micrococci/E. coli in 10% of samples. Caudry et al. Viridans Streptococci/E. coli in 17% of group I, 14% of group II, 10% of group III, and 15% of group IV [13]. Taji and Glass in their studies found widespread toothbrush contamination after its use except in cases where an oral antiseptic, such as mouthwash, was used instantly prior to brushing [15,16].

Warren et al. found that regular and triclosan-containing toothpaste use leads to lower toothbrush contamination than no toothpaste use [17]. Sato et al. found that rinsing toothbrushes just with tap water leads to continued elevated levels of contamination and biofilm [18]. Malmberg et al. isolated Staphylococcus epidermidis and Streptococci from toothbrushes after use while toothbrushes from both healthy patients and patients with oral disease developed potentially pathogenic bacteria such as Staphylococcus species, Pseudomonas, and E. coli species [19].

One of the limitations of the study is that further studies need to be conducted among different age groups and the results to be observed at different month intervals to have more understanding of contamination. Another limitation of the study was that we did not make a count on the number of colonies, if that had been done it would have provided the most strength to our study findings.

## Conclusions

In the present study, all toothbrushes were contaminated with different microorganisms when used for one month. The effectiveness of 0.2% chlorhexidine (34.17%), Listerine (30.84%), and Dettol (24.17%) were found to have a promising role but tap water (74.17%) was found to be ineffective. However, daily disinfection of toothbrushes and keeping it in a dry place better optimizes oral hygiene and systemic health.

## References

[1] Wade WG (2013). The oral microbiome in health and disease. Pharmacol Res.

[2] Glass RT, Lare MM (1986). Toothbrush contamination: a potential health risk?. Quintessence Int.

[3] Grewal N, Swaranjit K (1996). A study of toothbrush contamination at different time intervals and comparative effectiveness of various disinfecting solutions in reducing toothbrush contamination. J Indian Soc Pedod Prev Dent.

[4] Bunetel L, Tricot-Doleux S, Agnani G, Bonnaure-Mallet M (2000). In vitro evaluation of the retention of three species of pathogenic microorganisms by three different types of toothbrush. Oral Microbiol Immunol.

[5] Glass RT, Jensen HG (1994). The effectiveness of a u-v toothbrush sanitizing device in reducing the number of bacteria, yeasts and viruses on toothbrushes. J Okla Dent Assoc.

[6] Tomar P, Hongal S, Saxena V, Jain M, Rana K, Ganavadiya R (2014). Evaluating sanitization of toothbrushes using ultra violet rays and 0.2% chlorhexidine solution: a comparative clinical study. J Basic Clin Pharm.

[7] Frazelle MR, Munro CL (2012). Toothbrush contamination: a review of the literature. Nurs Res Pract.

[8] Sogi SH, Subbareddy VV, Kiran SN (2002). Contamination of toothbrush at different time intervals and effectiveness of various disinfecting solutions in reducing the contamination of toothbrush. J Indian Soc Pedod Prev Dent.

[9] Naik R, Ahmed Mujib BR, Telagi N, Anil BS, Spoorthi BR (2015). Contaminated tooth brushes-potential threat to oral and general health. J Family Med Prim Care.

[10] Ismaïl R, Aviat F, Michel V, Le Bayon I, Gay-Perret P, Kutnik M, Fédérighi M (2013). Methods for recovering microorganisms from solid surfaces used in the food industry: a review of the literature. Int J Environ Res Public Health.

[11] Osho A, Thomas BT, Akande YA, Udor RD (2013). Toothbrushes as fomites. J Dent Oral Hyg.

[12] Joy T, Venugopal S, Sadanandan S, Mathew M (2022). Evaluation of microbial contamination of toothbrushes and their decontamination using various disinfectants: n in vitro study. J Indian Assoc Public Health Dent.

[13] Caudry SD, Klitorinos A, Chan EC (1995). Contaminated toothbrushes and their disinfection. J Can Dent Assoc.

[14] Mehta A, Sequeira PS, Bhat G (2007). Bacterial contamination and decontamination of toothbrushes after use. N Y State Dent J.

[15] Taji SS, Rogers AH (1998). ADRF Trebitsch Scholarship. The microbial contamination of toothbrushes. A pilot study. Aust Dent J.

[16] Glass RT (1992). Toothbrush types and retention of microorganisms: how to choose a biologically sound toothbrush. J Okla Dent Assoc.

[17] Warren DP, Goldschmidt MC, Thompson MB, Adler-Storthz K, Keene HJ (2001). The effects of toothpastes on the residual microbial contamination of toothbrushes. J Am Dent Assoc.

[18] Sato S, Pedrazzi V, Guimarães Lara EH, Panzeri H, Ferreira de Albuquerque R Jr, Ito IY (2005). Antimicrobial spray for toothbrush disinfection: an in vivo evaluation. Quintessence Int.

[19] Malmberg E, Birkhed D, Norvenius G, Norén JG, Dahlén G (1994). Microorganisms on toothbrushes at day-care centers. Acta Odontol Scand.

